# The Influence of Psychological Ownership of Nature and Legacy Motivation on Pro-Environmental Decision-Making in the Intergenerational Dilemma

**DOI:** 10.3390/bs16050786

**Published:** 2026-05-15

**Authors:** Songyan Zha, Xiaoqi Li, Yuan Zhang

**Affiliations:** School of Psychology, Shaanxi Normal University, Xi’an 710062, China; zhahan1226@snnu.edu.cn (S.Z.); m15191273397@163.com (X.L.)

**Keywords:** psychological ownership of nature, legacy motivation, intergenerational dilemma, intergenerational goods game

## Abstract

Human behavior drives environmental degradation, yet research often neglects the intergenerational dimensions of pro-environmental decisions. Individuals frequently discount future generations’ needs due to power asymmetries, lack of reciprocity, and psychological distance, thereby exacerbating the tragedy of the commons. To address this intergenerational dilemma, this research investigates the roles of psychological ownership of nature and legacy motivation across two studies. The findings reveal that both constructs significantly promote intergenerational pro-environmental decision-making. Specifically, individuals with a stronger psychological connection to nature and higher impact legacy motivation are more likely to prioritize long-term environmental sustainability. Furthermore, impact legacy motivation mediates the effect of psychological ownership of nature on these decisions. Cultivating a sense of ownership over nature catalyzes a desire to leave a tangible positive impact, which subsequently drives sustainable choices. By translating environmental attachment into intergenerational responsibility, this study provides a robust empirical psychological foundation for policies aimed at fostering individual-level sustainable behaviors for future generations.

## 1. Introduction

The exacerbation of the global ecological crisis necessitates the implementation of arduous trade-offs between the satisfaction of contemporary needs and the protection of the interests of future generations. Nonetheless, when individuals engage in intertemporal decision-making, they often prioritize immediate self-interest. This incongruity between present self-interest and future interests of others represents a fundamental intergenerational dilemma (Wade-Benzoni & Tost, 2009). The presence of a marked power imbalance, an absence of reciprocal interactions, and psychological distance in intergenerational interactions can result in a high degree of susceptibility to self-serving biases. This, in turn, can lead to a systematic neglect of the interests of future generations (van Treek et al., 2023). Recent studies have confirmed that legacy motivation is a psychological mechanism that plays a vital role in resolving the intergenerational dilemma. The activation of an individual’s desire to leave a positive legacy has been shown to effectively shorten the intergenerational psychological distance, thereby significantly enhancing intergenerational cooperation and pro-environmental decision-making (Syropoulos et al., 2023b). Moreover, ecological and environmental issues are fundamentally dilemmas of public goods management, where the shared nature of resources such as air and water readily gives rise to a “tragedy of the commons.” The theory of psychological ownership offers a novel perspective for averting such tragedies. A substantial body of research suggests that this subjective, non-legal sense of ownership can considerably bolster individuals’ sense of responsibility toward public resources and promote pro-environmental decision-making, thereby effectively preventing resource depletion (Peck et al., 2021; Preston & Gelman, 2020). Psychological ownership of nature stems from control over a target, intimate knowledge, and self-investment, fundamentally acting as a contemporary extension of the self-boundary (Liang et al., 2024); meanwhile, legacy motivation achieves self-transcendence through the transmission of resources or values, shifting the decision-making perspective toward the future (Wade-Benzoni, 2019). When individuals develop a “this is mine/ours” perception of nature, why does this perception not evolve into an exclusionary endowment effect but instead transform into a legacy motivation to sacrifice present interests for future generations? This study argues that the key to resolving intergenerational conflicts lies in the unique attributes of the natural environment and the self-construal inherent in collectivistic cultures. First, the natural environment is characterized by non-exclusivity and intergenerational shareability. Second, within Chinese collectivistic culture, the concept of “us” is notably inclusive and expansive. When individuals perceive nature as an extended self while facing the inherent finitude of life, transmitting this extended self to future generations becomes a primary way to maintain self-continuity and existential meaning. Integrating the unique roles of legacy motivation and psychological ownership of nature in intertemporal decision-making, this study explores how these variables independently or synergistically drive pro-environmental choices. By examining these psychological mechanisms, we aim to provide new theoretical insights and empirical evidence to overcome intergenerational dilemmas and promote ecological sustainability.

### 1.1. Pro-Environmental Decision-Making

Environmental issues are fundamentally rooted in human decision-making. Pro-environmental decision-making refers to the choices individuals make to improve the ecological environment or mitigate environmental damage. This study posits that such decision-making encompasses both active environmental protection and the reduction of destructive behaviors, centering on how individuals weigh the potential ecological impacts of various options. The primary objective of research in this field is to elucidate the psychological mechanisms and contextual factors driving individual behavior. The existing literature broadly categorizes these influential factors into personal (e.g., personality, values, cognitive biases) and social (e.g., norms, culture, social class) dimensions (Gifford & Nilsson, 2014). Studies have found that the trade-off between behavioral costs and environmental benefits plays a crucial moderating role: High temporal or financial costs often inhibit pro-environmental choices (Kramer & Petzoldt, 2022), whereas low-cost contexts significantly promote such behaviors (Wyss et al., 2023). However, when high costs are effectively offset by potential gains or environmental benefits, individuals’ pro-environmental tendencies often increase (Li et al., 2023; Yamawaki et al., 2023). Beyond cost considerations, social interaction and reputation management are also crucial determinants; research indicates that compared to anonymous contexts, observability significantly enhances pro-environmental behavior toward both acquaintances and strangers, primarily driven by the need to maintain social reputation (Zhong et al., 2022). Fundamentally, pro-environmental decision-making is often viewed as a social dilemma, a conflict between individual rationality and collective rationality (Zhang et al., 2022). Klein et al. (2022) noted that intergroup conflicts of interest and disparities in resource acquisition hinder cooperation, whereas reducing social distance promotes pro-environmental behavior. To quantify these complex psychological processes, researchers widely employ the greater good game (GGG), the dictator game (DG), and donation paradigms as measurement tools (Chan et al., 2024; Chen & Krajbich, 2018; Klein et al., 2017). These paradigms effectively reveal the underlying motivations and psychological mechanisms involved in balancing resource allocation and environmental protection.

### 1.2. Intergenerational Dilemma

Sustainable development is not only a fundamental goal for societal progress but also requires decision-makers to possess a forward-looking perspective that transcends the present. However, such proactive decision-making often necessitates sacrificing current interests for the welfare of future generations; this trade-off frequently leads to an intergenerational dilemma. The intergenerational dilemma refers to the zero-sum game encountered by current decision-makers between their immediate personal interests and the well-being of future generations (Wade-Benzoni & Tost, 2009). The essence of this dilemma lies in the asymmetry of power and time: The preceding generation possesses unilateral decision-making power, while future generations can neither participate in the decision-making process nor enforce retroactive accountability (Wade-Benzoni & Tost, 2009). Furthermore, the delayed and uncertain nature of these outcomes further exacerbates the difficulty of intergenerational cooperation (Wade-Benzoni & Tost, 2009). Concurrently, the delayed and uncertain nature of these decisions further exacerbates the difficulty of intergenerational cooperation (van Treek et al., 2023). To mitigate this dilemma, existing research has primarily focused on shortening the psychological distance between generations and exploring mechanisms to promote intergenerational cooperation through cognitive and motivational pathways. At the cognitive level, interventions such as perspective-taking, introducing intergenerational accountability, and establishing future-representative voting systems prompt decision-makers to integrate the perspectives of future generations into their cognitive frameworks, thereby enhancing their propensity for pro-environmental decision-making (Kamijo et al., 2019; Katsuki & Hizen, 2020; Saijo, 2020; Shahen et al., 2020, 2021; Timilsina et al., 2023). At the psychological and affective level, legacy motivation has been proven to be a core factor driving intergenerational altruistic behavior (Wade-Benzoni, 2019). Intergenerational reciprocity plays a key role here: When individuals perceive that they have inherited resources from preceding generations or reflect on the sacrifices made by their predecessors, feelings of gratitude stimulate their sense of responsibility toward future generations, thereby increasing their inclination to make sustainable decisions (Chang et al., 2021; Syropoulos et al., 2020; Watkins & Goodwin, 2020). However, if the decisions of preceding generations failed to yield long-term benefits, reflecting on the past may be insufficient. In such cases, adopting a future-oriented perspective, by predicting future behaviors or eliciting concern for the survival of descendants, can more effectively facilitate intergenerational cooperation (Bosetti et al., 2022; Liu et al., 2024). Regarding research paradigms, scholars primarily examine intergenerational decision-making through scenario simulations and gaming tasks. While scenario simulations are easy to implement due to their operational simplicity, they often capture hypothetical intentions rather than consequential behavior. In contrast, the intergenerational goods game (IGG) and the intergenerational sustainability dilemma game (ISDG), which are adaptations of the public goods game, offer higher ecological validity. These paradigms construct a resource chain with intergenerational transmission effects, making the payoffs for “future others” directly contingent upon the decisions of the preceding generation, thereby more accurately simulating the dynamic processes and psychological mechanisms of intergenerational resource allocation in the real world.

### 1.3. Psychological Ownership of Nature

Psychological ownership is derived from the legal concept of ownership and is defined as an individual’s subjective psychological state of feeling that a target object, or a portion of it, is “mine” or “ours” (Pierce et al., 1991). This concept has been widely applied in organizational management, consumer behavior, and natural environments (Avey et al., 2009; Morewedge, 2021; She et al., 2022). Grounded in China’s collectivistic culture and the public goods attribute of the environment, this research focuses specifically on collective psychological ownership of nature, defined as the psychological perception whereby individuals perceive the natural environment as ours. Psychological ownership of nature exerts a dual effect: On the one hand, collective ownership can significantly enhance individuals’ shared sense of responsibility for environmental protection and effectively promote pro-environmental behaviors; on the other hand, it may also trigger territoriality in certain contexts. (Martinović & Verkuyten, 2024). Furthermore, excessive or exclusionary individual ownership can activate territoriality, inducing dominating and utilitarian attitudes that lead individuals to exploit rather than protect nature (Martinović & Verkuyten, 2024; Ross et al., 2018). Although psychological ownership may involve maladaptive aspects, such as triggering territoriality and exclusive possession, it is more likely to induce a stewardship mindset within environmental contexts and collectivistic cultures. Unlike private goods, the natural environment cannot be permanently physically possessed by an individual. Therefore, the sense of control elicited by collective psychological ownership of nature is directed toward responsibility rather than exploitation. Particularly when our identity is extended temporally, protecting the environment becomes a necessary means of preserving an intergenerational common asset. Building upon this, the present study integrates psychological ownership of nature into the framework of the intergenerational dilemma, proposing Hypothesis 1: Collective psychological ownership of nature will significantly promote pro-environmental decision-making in the intergenerational dilemma.

### 1.4. Legacy Motivation

Legacy motivation provides a central perspective for explaining how individuals make intertemporal decisions within the intergenerational dilemma. Also referred to as the transmission motive, legacy motivation is defined as an individual’s psychological desire to exert a long-term impact on others through their actions, thereby transcending their mortal timeline and imbuing their personal identity with enduring meaning (Wade-Benzoni & Tost, 2009; Zaval et al., 2015). Syropoulos et al. (2023b) distinguished this construct into impact legacy motivation, which focuses on substantive contributions, and reputation legacy motivation, which focuses on being remembered positively by future generations. The present study adopts the overarching definition of legacy motivation proposed by Zaval et al. (2015) and incorporates the dimensional framework of Syropoulos et al. (2023b) for mechanism testing. The psychological foundation of legacy motivation is rooted in terror management theory; specifically, mortality salience prompts individuals to develop a desire to leave a positive legacy as a buffer against concerns regarding the brevity of life (Burke et al., 2010; Sligte et al., 2013; Syropoulos & Markowitz, 2021; Wickersham et al., 2020). Legacy motivation plays a critical role in promoting intergenerational cooperation, significantly enhancing individuals’ sustainable decisions, behaviors, and attitudes in the environmental domain (Bang et al., 2017; Hurlstone et al., 2020; Wickersham et al., 2020). However, the efficacy of legacy motivation is subject to a critical boundary condition: Individuals must perceive that their actions can effectively alter future environmental outcomes (Wade-Benzoni, 2019). We propose that psychological ownership of nature serves as the crucial antecedent providing this perceived control. By internalizing the well-being of the natural environment as an extension of the self (Liang et al., 2024), individuals develop the belief that they possess the capacity to influence and maintain ecological balance, thereby activating legacy motivation. Based on this, we propose the following hypotheses. Hypothesis 2: Legacy motivation promotes individuals’ pro-environmental decision-making in the intergenerational dilemma. Hypothesis 3: Psychological ownership of nature influences pro-environmental decision-making in the intergenerational dilemma by affecting legacy motivation. In terms of legacy motivation research, studies have primarily utilized writing tasks to prime this construct, often supplementing them with scale-based measurements (Zaval et al., 2015). Recently, the examination of distinct sub-dimensions has gained prominence (Syropoulos et al., 2023b); investigating these dimensions separately not only enhances measurement precision but also helps independently test the influence mechanisms of each dimension on intergenerational pro-environmental decision-making. Therefore, this study proposes Hypothesis 4: Impact legacy motivation and reputation legacy motivation mediate the relationship between psychological ownership of nature and pro-environmental decision-making in the intergenerational dilemma.

### 1.5. Current Research

This study aims to systematically explore the mechanisms through which psychological ownership of nature and legacy motivation influence pro-environmental decision-making within the framework of the intergenerational dilemma. To this end, a multi-method approach is employed to strengthen the reliability of the findings and the rigor of causal inference. Study 1 adopts a survey-based study to examine the relationships among psychological ownership of nature, the dimensions of legacy motivation, and pro-environmental decision-making in an intergenerational context. Structural equation modeling (SEM) is utilized to construct and validate the structural relationships among these variables. Building on the model established in Study 1, Study 2 utilizes the intergenerational goods game (IGG) paradigm to create a realistic intergenerational dilemma scenario. Through the experimental manipulation of psychological ownership of nature and legacy motivation, Study 2 further tests and elucidates the causal pathways and mediating effects within the proposed model.

## 2. Study 1

### 2.1. Research Objective

This study aims to investigate the relationships among psychological ownership of nature, legacy motivation, and intergenerational pro-environmental decision-making. It examines the direct predictive role of psychological ownership of nature on decision-making and elucidates the mediating effects of legacy motivation, specifically focusing on its impact and reputation dimensions, to test Hypotheses 1 through 4.

### 2.2. Method

#### 2.2.1. Participants

Participants were recruited via the Credamo platform. We recruited 505 participants (*M_age_* = 24.92, *SD_age_* = 5.93), including 249 males (49.3%) and 256 females (50.7%). A total of 71.3% held a bachelor’s degree, and 18.8% held a master’s degree, with 75.2% residing in urban areas and 24.8% in rural areas.

#### 2.2.2. Materials

Collective psychological ownership of nature scale (CPON): The collective psychological ownership of nature scale (CPON) was developed by X. Wang et al. (2023) to measure collective psychological ownership of nature. Translated and revised for the Chinese context, the scale comprises one dimension labeled “Sense,” with seven items rated on a seven-point Likert scale (1 = strongly disagree, 7 = strongly agree). Higher scores reflect a stronger psychological perception that “nature belongs to us.” Cronbach’s alpha for this scale was 0.92.

Legacy motives scale (LM): The legacy motives scale (LM) was constructed by Syropoulos et al. (2023b) based on previous measurement items. Translated and revised for the Chinese context, the scale includes two dimensions: Impact legacy motives (ILMs) and reputation legacy motives (RLMs), with four items for each dimension (eight items in total). All items were scored on a seven-point Likert scale (1 = strongly disagree, 7 = strongly agree). Higher scores indicate higher levels of legacy motives. Cronbach’s alpha for this scale was 0.90.

Pro-environmental decision-making in the intergenerational dilemma: Pro-environmental decision-making in the intergenerational dilemma was measured using the donation task developed by Zaval et al. (2015), translated and revised for the Chinese context. The scenario highlighted the well-being of future generations to embed an intergenerational focus. Participants were asked to allocate their 10-yuan participation fee between donation and retention. The amount donated was used as the indicator of pro-environmental decision-making.

### 2.3. Results

#### 2.3.1. Correlation Analysis

The results indicated that psychological ownership of nature was significantly and positively correlated with pro-environmental decision-making in the intergenerational dilemma (*r* = 0.16, *p* < 0.001). It also showed a significant positive correlation with legacy motivation (*r* = 0.37, *p* < 0.001) and its two dimensions: impact legacy motivation (*r* = 0.47, *p* < 0.001) and reputation legacy motivation (*r* = 0.23, *p* < 0.001). Furthermore, legacy motivation (*r* = 0.24, *p* < 0.001) and its two dimensions—impact legacy motivation (*r* = 0.28, *p* < 0.001) and reputation legacy motivation (*r* = 0.17, *p* < 0.001)—were significantly and positively correlated with pro-environmental decision-making in the intergenerational dilemma (see Table 1).

#### 2.3.2. Mediation Analysis

The mediation effect was tested using the Process macro. Regression analysis results (see Table 2) indicated that psychological ownership of nature significantly and positively predicted pro-environmental decision-making in the intergenerational dilemma (*β* = 0.16, *t* = 3.58, *p* < 0.001) and significantly and positively predicted legacy motivation (*β* = 0.37, *t* = 9.09, *p* < 0.001); legacy motivation significantly and positively predicted pro-environmental decision-making in the intergenerational dilemma (*β* = 0.23, *t* = 4.79, *p* < 0.001), supporting Hypotheses 1 and 2.

Further mediation analysis using 5000 bootstrap samples (see Table 3) revealed that legacy motivation played a significant mediating role between psychological ownership of nature and pro-environmental decision-making in the intergenerational dilemma, *b* = 0.08, *SE* = 0.02, 95% CI [0.05, 0.13], supporting Hypothesis 3.

#### 2.3.3. The Mediating Effect of Impact Legacy Motivation

**Figure 1:**
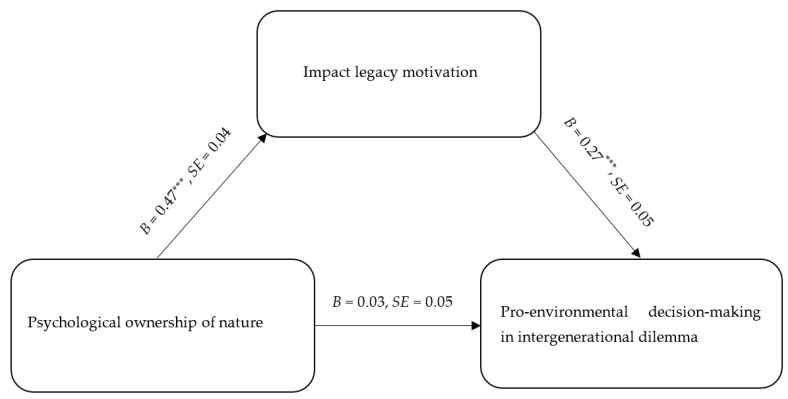
Mediating model diagram of factors impacting legacy motivation (*N* = 505). Note: *** *p* < 0.001.

Results demonstrated that impact legacy motivation played a significant mediating role between psychological ownership of nature and pro-environmental decision-making in the intergenerational dilemma (see Table 3, Figure 1), *b* = 0.11, *SE* = 0.03, 95% CI [0.06, 0.18], whereas the mediating effect of reputation legacy motivation was not significant, *b* = 0.01, *SE* = 0.01, 95% CI [−0.01, 0.04], Thus, Hypothesis 4 was partially supported.

### 2.4. Robustness Tests

To validate the mediation results obtained via the PROCESS macro and ensure the methodological robustness of our findings, we re-estimated the proposed mediation model using structural equation modeling (SEM) with the lavaan package in R 4.5.3. The SEM path analysis utilized 5000 bootstrap resamples to calculate the 95% bias-corrected confidence intervals for the indirect effects. Consistent with the primary analysis, the SEM results confirmed that the indirect effect of psychological ownership of nature on pro-environmental decision-making via impact legacy motivation remained highly significant (standardized indirect effect = 0.115, 95% CI [0.163, 0.502]). In contrast, the indirect path through reputation legacy motivation was non-significant (standardized indirect effect = 0.011, 95% CI [−0.039, 0.101]).

Furthermore, after controlling for covariates such as gender and age, the structural paths consistently showed that psychological ownership of nature significantly predicted impact legacy motivation (*β* = 0.472, *p* < 0.001), and impact legacy motivation significantly predicted pro-environmental decision-making (*β* = 0.244, *p* < 0.001).

### 2.5. Discussion

This study utilized a survey-based approach to construct a structural equation model, providing support for all core hypotheses. First, psychological ownership of nature significantly promoted pro-environmental decision-making within the intergenerational dilemma, a finding highly consistent with previous research (Martinović & Verkuyten, 2024). Second, psychological ownership of nature significantly and positively predicted legacy motivation. While prior studies focused on how structural power asymmetry (e.g., the decision-maker’s role) activates legacy motivation (Tost et al., 2015), the present study extends this paradigm to the psychological level. We found that the sense of control and perceived power derived from psychological ownership similarly activate legacy motives. Once individuals conceptualize nature as an extended self, the motivation to protect the environment and bequeath a sustainable ecosystem becomes coupled with the need to buffer mortality anxiety and achieve symbolic immortality (Wade-Benzoni & Tost, 2009), thereby inspiring a willingness to leave a positive environmental legacy. Furthermore, this study revealed dimensional differences: psychological ownership of nature promoted pro-environmental decisions by stimulating tangible impact legacy motivation. Conversely, the mediating role of reputation legacy motivation was not supported, likely because the anonymous donation paradigm lacked the social surveillance and incentive contexts necessary for reputation management. This aligns with findings in public goods games (J. Wang & Dai, 2020), further demonstrating the context-dependency of different legacy motivation dimensions.

## 3. Study 2

Study 1 established the correlational relationships among psychological ownership of nature, legacy motivation, and pro-environmental decision-making within the intergenerational dilemma. To further elucidate the causal pathways between these variables and validate the mediating mechanisms, Study 2 comprised two experiments. Experiment 1 manipulated psychological ownership of nature to test its causal impact on legacy motivation and pro-environmental decision-making; Experiment 2 manipulated legacy motivation to test its direct effect on decision-making.

### 3.1. Experiment 1: The Impact of Psychological Ownership of Nature on Legacy Motivation and Intergenerational Pro-Environmental Decision-Making

#### 3.1.1. Pre-Test: Validation of the Manipulation Materials for Psychological Ownership of Nature

To verify the effectiveness of the manipulation materials for psychological ownership of nature, we recruited 128 participants (*M_age_* = 30.12, *SD_age_* = 8.39), including 49 males (38.3%) and 79 females (61.7%). A total of 71.9% held a bachelor’s degree, and 14.1% held a master’s degree, with 85.9% residing in urban areas and 24.8% in rural areas. Participants were randomly assigned to either the priming group or the control group. After reading the manipulation or control materials, they completed the collective psychological ownership of nature scale. The priming group read natural description materials that emphasized the use of the first-person plural pronoun “our” (adapted from Preston & Gelman, 2020), while the control group read neutral descriptive materials. A one-way ANOVA revealed a significant difference in scores: The priming group’s scores (*M* = 5.85, *SD* = 0.52) were significantly higher than those of the control group (*M* = 5.38, *SD* = 1.09), *F* (1, 126) = 9.89, *p* < 0.01, *ηp*^2^ = 0.073. These results indicate that the experimental manipulation was successful.

#### 3.1.2. Formal Experiment: The Impact of Psychological Ownership of Nature on Legacy Motivation and Pro-Environmental Decision-Making in the Intergenerational Dilemma

(1) Objective

This experiment examined the impact of psychological ownership of nature on legacy motivation and intergenerational pro-environmental decision-making through its manipulation.

(2) Design

A single-factor (psychological ownership of nature: priming vs. control) between-subjects design was adopted. The dependent variables were scores on the legacy motivation scale and the number of tokens donated to the forest collective account in the intergenerational goods game (IGG).

(3) Method

① Participants

To ensure sufficient statistical power, the required sample size was determined using G*Power 3.1.9.7. The power analysis indicated a minimum total sample size of 172 participants, 86 participants per group. (1 − *β* = 0.90, *α* = 0.05, *f* = 0.25). A total of 180 valid participants were recruited via the Credamo platform (*M_age_* = 31.01, *SD_age_* = 7.08), including 80 males (44.4%) and 100 females (55.6%). A total of 62.2% held a bachelor’s degree, and 27.8% held a master’s degree, with 90.6% residing in urban areas and 9.4% in rural areas. Participants were randomly assigned to two groups, with 90 in each.

② Materials

Manipulation of psychological ownership of nature: The manipulation materials were identical to those used in the pre-test.

Measurement instruments: The collective psychological ownership of nature scale and the two-dimensional legacy motivation scale were employed.

**Figure 2:**
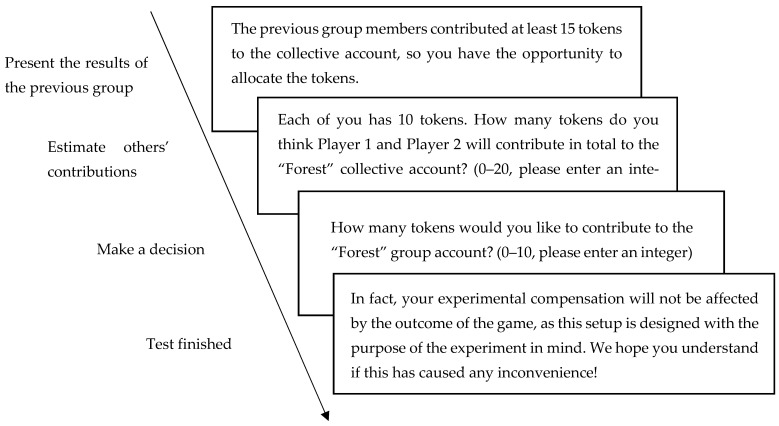
Intergenerational goods game flowchart.

**Figure 3:**
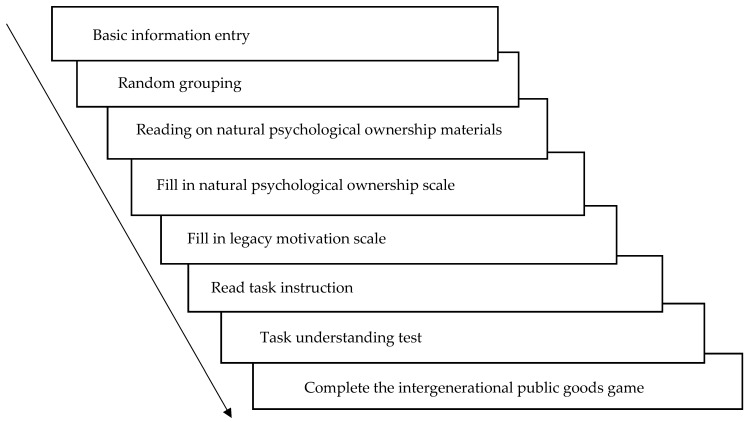
Experimental flowchart.

Intergenerational goods game (IGG) paradigm: Pro-environmental decision-making in the intergenerational dilemma was measured using the IGG paradigm under a “give” frame (Lohse & Waichman, 2020). Participants were grouped with two virtual players to form the “current generation”. Each player was endowed with 10 initial tokens and had to decide how many tokens to allocate to their private account (converted to personal payoff at a 1:1 ratio) versus the “forest” collective account. If the total contributions to the collective account reached or exceeded a threshold of 15 tokens, the “future generation” of players would receive resources to continue the game; otherwise, the game ended for all subsequent generations. The amount contributed to the collective account served as the behavioral indicator of pro-environmental decision-making. See Figure 2. The experimental procedure is illustrated in Figure 3.

(4) Results

① Manipulation Check

The experimental group’s psychological ownership of nature scores (*M* = 5.93, *SD* = 0.58) were significantly higher than the control group’s (*M* = 5.23, *SD* = 1.19), *F* (1, 178) = 24.87, *p* < 0.001, *ηp*^2^ = 0.123, indicating a successful manipulation.

② Effect on Legacy Motivation

For impact legacy motivation, a one-way ANOVA revealed a significant main effect of psychological ownership of nature; the experimental group scored significantly higher (*M* = 5.99, *SD* = 0.47) than the control group (*M* = 5.78, *SD* = 0.71), *F* (1, 178) = 5.49, *p* < 0.05, *ηp*^2^ = 0.030. This main effect remained significant after controlling for gender and age, *F* (1, 176) = 6.58, *p* < 0.05, *ηp*^2^ = 0.036.

Reputation legacy motivation (see Table 4): The main effect of psychological ownership of nature was not significant. No significant difference was observed between the experimental group (*M* = 5.40, *SD* = 0.90) and the control group (*M* = 5.28, *SD* = 1.05), *F* (1, 178) = 0.64, *p* > 0.05, *ηp*^2^ = 0.004.

③ The Impact of Psychological Ownership of Nature on Pro-environmental Decision-making in the Intergenerational Dilemma

One-way ANOVA results (see Table 4) revealed a significant main effect of psychological ownership of nature. Participants in the experimental group (*M* = 6.74, *SD* = 1.50) exhibited significantly higher levels of pro-environmental decision-making in the intergenerational dilemma context compared to the control group (*M* = 6.30, *SD* = 1.47), *F* (1, 178) = 4.02, *p* < 0.05, *ηp*^2^ = 0.022. After controlling for gender and age, the main effect of psychological ownership of nature remained significant, *F* (1, 176) = 4.00, *p* < 0.05, *ηp*^2^ = 0.022.

④ Mediation Analysis

**Figure 4:**
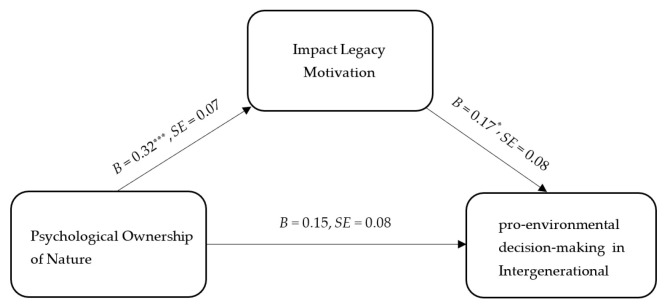
Mediating model diagram of factors impacting legacy motivation (*N* = 180). Note: * *p* < 0.05, *** *p* < 0.001.

The mediation effect was tested using the PROCESS macro for SPSS 26.0. The path coefficients are illustrated in Figure 4. Mediation analysis results based on 5000 bootstrap samples indicated that the indirect effect of impact legacy motivation was significant, *b* = 0.05, *SE* = 0.03, 95% CI [0.008, 0.130]. However, the mediating role of reputation legacy motivation was not supported. These findings provide further empirical support for Hypothesis 4.

### 3.2. Experiment 2: The Impact of Legacy Motivation on Pro-Environmental Decision-Making in the Intergenerational Dilemma

#### 3.2.1. Pre-Test: Evaluation of Legacy Motivation Manipulation Materials

To verify the effectiveness of the legacy motivation manipulation materials, 48 participants were recruited (*M_age_* = 30.81, *SD_age_* = 7.20), including 23 males (47.92%) and 25 females (52.08%). A total of 68.75% held a bachelor’s degree, and 25% held a master’s degree, with 87.5% residing in urban areas and 12.5% in rural areas. Participants were randomly assigned to one of three groups: the impact legacy motivation group, the reputation legacy motivation group, or the control group. The manipulation was conducted via a structured writing task (Syropoulos et al., 2023a).

One-way ANOVA results indicated that the scores for the impact legacy motivation priming group (*M* = 6.17, *SD* = 0.35) were significantly higher than those of the control group (*M* = 5.58, *SD* = 0.99), *p* < 0.05, confirming the effectiveness of the impact legacy manipulation. Crucially, the impact legacy manipulation did not cause significant changes in reputation legacy motivation. For the reputation legacy motivation priming group, scores (*M* = 5.98, *SD* = 0.46) were significantly higher than both the impact legacy group (*M* = 5.34, *SD* = 0.54), *p* < 0.05, and the control group (*M* = 5.41, *SD* = 1.03), *p* < 0.05, indicating that the reputation legacy motivation manipulation was also effective. Furthermore, this manipulation was independent and did not affect impact legacy motivation. This double dissociation demonstrates that the dimensional manipulation of legacy motivation is both effective and independent.

#### 3.2.2. Formal Experiment: The Impact of Legacy Motivation on Pro-Environmental Decision-Making in the Intergenerational Dilemma

(1) Research Objective

This experiment aimed to examine the causal impact of legacy motivation on pro-environmental decision-making within an intergenerational dilemma context by manipulating the two dimensions of legacy motivation.

(2) Experimental Design

A single-factor (legacy motivation: impact group vs. reputation group vs. control group) between-subjects design was employed. The dependent variable was the number of tokens contributed to the “forest” collective account in the intergenerational goods game (IGG).

(3) Method

① Participants and materials

Based on G*Power 3.1.9.7, the required sample size was calculated to be 207 participants (1 − *β* = 0.90, *α* = 0.05, *f* = 0.25). A total of 230 valid participants were recruited via the Credamo platform. (*M_age_* = 29.40, *SD_age_* = 8.09), including 98 males (42.61%) and 132 females (57.39%). A total of 69.57% held a bachelor’s degree, and 16.52% held a master’s degree, with 84.78% residing in urban areas and 15.22% in rural areas. Participants were randomly assigned to the impact group (*n* = 79), the reputation group (*n* = 75), and the control group (*n* = 76).

Manipulation materials: The legacy motivation writing task was identical to that used in the pre-test.

Measurement scale: The two-dimensional legacy motivation scale was utilized.

Decision-making paradigm: The IGG paradigm from Experiment 1 was employed.

**Figure 5:**
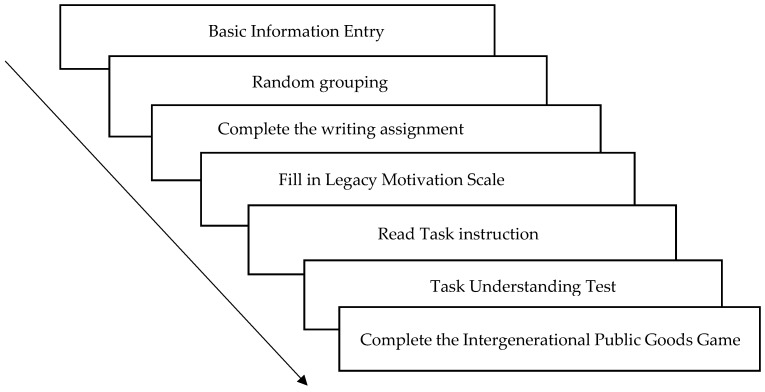
Experimental flowchart.

② The experimental procedure is detailed in Figure 5.

(4) Results

① Manipulation Check

The impact legacy motivation group (*M* = 5.91, *SD* = 0.58) scored significantly higher than the control group (*M* = 5.55, *SD* = 0.80), *p* < 0.01, confirming the effectiveness of the impact manipulation. Similarly, the reputation legacy motivation group (*M* = 5.65, *SD* = 0.84) scored significantly higher than the control group (*M* = 5.04, *SD* = 0.97), *p* < 0.001, confirming the effectiveness of the reputation manipulation.

② The Influence of Legacy Motivation on Pro-environmental Decision-making

One-way ANOVA revealed a significant main effect of legacy motivation on pro-environmental decision-making, *F* (2, 227) = 5.74, *p* = < 0.01, *ηp*^2^ = 0.048 (see Table 5). After controlling for gender and age, the significant difference persisted, *F* (2, 225) = 6.01, *p* < 0.01, *ηp*^2^ = 0.051. Further post hoc tests indicated that the impact legacy motivation group (*M* = 6.48, *SD* = 1.58) exhibited significantly higher levels of pro-environmental decision-making than the control group (*M* = 5.67, *SD* = 1.16), *p* < 0.01. This difference remained robust after controlling for gender and age, *F* (1, 151) = 13.75, *p* < 0.001, *ηp*^2^ = 0.083. However, no significant difference was found between the reputation legacy motivation group (*M* = 6.01, *SD* = 1.70) and the control group (*M* = 5.67, *SD* = 1.16), *p* > 0.05.

### 3.3. Robustness Tests

Given that the dependent variable (token allocation) in the intergenerational public goods game was measured as a discrete variable ranging from 0 to 10, we conducted non-parametric tests to confirm the robustness of the ANOVA results. Specifically, for Experiment 1, a Mann–Whitney U test confirmed that participants in the psychological ownership of nature priming group allocated significantly more tokens to the collective account (mean rank = 99.29) than those in the control group (mean rank = 81.71), *Z* = −2.34, *p* = 0.019. This non-parametric outcome is entirely consistent with the primary parametric analysis, indicating that the main effect is robust against potential violations of the normality assumption.

Similarly, for Experiment 2, a Kruskal–Wallis H test was conducted to compare token allocations among the impact legacy motivation group, the reputation legacy motivation group, and the control group. The test revealed a significant overall difference in token contributions across the three groups, χ^2^(2) = 8.57, *p* = 0.014. This non-parametric result strongly corroborates the significant main effect found in the primary ANOVA, further demonstrating that our findings regarding legacy motivation are robust and not artifacts of the data distribution.

### 3.4. Discussion

Experiments 1 and 2 successfully validated the causal chain of the mediation model through double-randomized manipulations of psychological ownership of nature and legacy motivation. The experimental results were highly consistent with Study 1, reconfirming that impact legacy motivation mediated the relationship between psychological ownership of nature and intergenerational pro-environmental decision-making, while the mediating effect of reputation legacy motivation was not supported. Experiment 1 confirmed that psychological ownership of nature significantly promoted impact legacy motivation but had no obvious effect on reputation legacy motivation. This finding suggests that the sense of control and responsibility generated by psychological ownership of nature is more inclined to activate individuals’ intrinsic altruistic tendencies and desire for actual positive impact (Jami et al., 2021) rather than external reputation pursuit, thereby expanding research on the scope of altruistic behaviors associated with psychological ownership of nature. Experiment 2 further verified that only impact legacy motivation significantly promoted intergenerational pro-environmental decision-making. In summary, by viewing the natural environment as an “extended self” (Pierce & Jussila, 2010), individuals acquire psychological control over natural resources and form asymmetric decision-making power over future generations. This sense of power substantially enhances the contemporary generation’s sense of responsibility and altruistic tendencies (Cai et al., 2016), ultimately crystallizing into a need for impact legacy motivation and driving individuals to make pro-environmental decisions at the expense of their self-interest. Reputation legacy motivation failed to demonstrate a significant driving effect in the model, likely due to its sensitivity to the delayed nature of intergenerational feedback and the constraints of the anonymous experimental design.

## 4. Discussion

### 4.1. The Impact of Psychological Ownership of Nature on Intergenerational Pro-Environmental Decision-Making

The present study extends the research perspective of psychological ownership of nature from a traditional parallel intragenerational dimension to an intergenerational conflict dimension. Previous research has established that psychological ownership of nature can enhance intragenerational responsibility and foster pro-environmental behavior (Mishra et al., 2023; Preston & Gelman, 2020; She et al., 2022). The current findings further corroborate its efficacy within the context of intergenerational conflicts of interest. The facilitating effect of psychological ownership of nature primarily stems from the expansion of self-boundaries: When individuals develop psychological ownership over nature, the environment is perceived as an “extended self” (Pierce & Jussila, 2010), which cognitively generates a normative pressure that “we have a responsibility to take care of what is ours” (Verkuyten & Martinovic, 2017). This heightened collective responsibility and normative pressure increase individuals’ willingness to sacrifice immediate interests when navigating trade-offs between present and future benefits, thereby protecting the ecological environment as an intergenerational common asset. Consequently, psychological ownership of nature not only strengthens individual responsibility but also serves as an effective psychological lever to mitigate the “tragedy of the commons” and “free-rider effects” by elevating collective responsibility.

### 4.2. The Mediating Role of Legacy Motivation

Mediation analysis reveals that legacy motivation is the key endogenous mechanism through which psychological ownership of nature promotes pro-environmental decision-making in intergenerational dilemmas. Specifically, impact legacy motivation serves as the sole mediator for the indirect effect of psychological ownership of nature, whereas reputation legacy motivation shows no significant effect.

#### 4.2.1. The Impact of Psychological Ownership of Nature on Legacy Motivation

This study found that psychological ownership of nature significantly promotes altruistically oriented impact legacy motivation rather than egocentric reputation legacy motivation. The sense of psychological control and ownership derived from this construct conceptually endows the present generation with asymmetric decision-making power over resources. Similar to structural power (Tost et al., 2015), this psychological power elicits the moral sense and altruistic tendencies of the current generation (Jami et al., 2021), compelling individuals to focus on generating a positive impact for future descendants. By transforming the natural environment into a vessel of the “extended self”, protecting the environment ensures the preservation of this self-extension in the future, thereby achieving “symbolic immortality” (Wade-Benzoni & Tost, 2009). This intrinsic motivation for self-continuity drives high levels of impact legacy motivation. In contrast, reputation legacy motivation focuses on self-image maintenance; however, because the efficacy and power derived from psychological ownership of nature do not rely on external evaluation, its role in stimulating reputation concerns remains negligible (Imada et al., 2023).

#### 4.2.2. The Impact of Legacy Motivation on Pro-Environmental Decision-Making in the Intergenerational Dilemma

Study 2 found that impact legacy motivation significantly promotes pro-environmental decision-making within the intergenerational dilemma, which is consistent with existing research (Bang et al., 2017; Hurlstone et al., 2020; Wickersham et al., 2020). The consequences of intergenerational decisions are inherently delayed (van Treek et al., 2023); feedback and evaluations from future generations may take decades to materialize, which profoundly weakens the utility of decisions driven by external reputation. Furthermore, the anonymous design employed in this study eliminated social surveillance, further depriving reputation motives of the prerequisites necessary to exert their effect (J. Wang & Dai, 2020). In contrast, impact legacy motivation focuses on tangible altruistic outcomes, making it more conducive to intergenerational environmental decisions that require the present generation to sacrifice immediate interests to transmit a habitable environment to future descendants.

### 4.3. Theoretical Contributions and Practical Implications

#### 4.3.1. Theoretical Contributions

Theoretically, this study examines the effects of psychological ownership of nature and legacy motivation on pro-environmental decision-making within an intergenerational dilemma framework. It provides an empirical psychological foundation for intergenerational environmental policies, facilitating the translation of sustainability goals into individual-level implementation. Furthermore, it expands the theories of psychological ownership and legacy motivation by elucidating their distinct roles in intergenerational decision-making.

#### 4.3.2. Practical Implications

From a practical perspective, future environmental protection efforts should focus on the following strategies. Empowerment strategies for psychological ownership of nature: Environmental communication should reframe narratives from “the environment” to “our nature”, employing first-person plural pronouns to strengthen collective ownership. At the policy level, promoting participatory governance, such as community gardens and ecological patrols, can enhance residents’ sense of ownership by granting them tangible control over specific natural areas. Activation of impact legacy motivation: Utilizing intergenerational narratives, such as “Your environmental choices today determine the quality of life for your descendants a century from now”, can elevate individuals’ awareness of their intergenerational impact. In educational settings, immersive VR technology or activities like writing long-term environmental pledges can help internalize the responsibility of leaving a positive ecological legacy. Symbolic guardianship mechanisms: Promoting symbolic guardianship rights, such as “ecological legacy certificates”, can reify environmental responsibility and endow it with intergenerational inheritability. This transforms ecological stewardship into a transmissible intergenerational contract, thereby reinforcing altruistic behavior toward future generations.

### 4.4. Policy Implications

First, macro-level environmental governance structures should embrace appropriate decentralization, transitioning from “top-down administrative mandates” to “bottom-up community co-management.” Governments could implement policies to delegate tangible stewardship and daily management rights of specific natural resources—such as community parks, local watersheds, and peri-urban forests—to local residents. By granting the public substantive environmental management authority, policymakers can firmly establish collective psychological ownership of nature at the institutional level without altering legal property rights. This approach fosters a robust psychological perception of “our nature,” thereby intrinsically driving collective pro-environmental decision-making and internalizing pro-environmental behaviors.

Second, the communication of environmental policies should be structurally aligned with impact legacy motivation. Traditional environmental economic policies frequently focus on short-term interests, and public awareness campaigns often rely on appeals to immediate threats or short-term financial incentives. Our findings suggest that at the policy level, environmental protection should be reframed as a meaningful intergenerational contribution. Governments should innovate green finance and incentive mechanisms, such as establishing “intergenerational ecological trusts” or issuing “green legacy bonds.” These instruments would allow individuals and corporations to invest in ecological restoration projects with cycles spanning decades or even centuries, while policy frameworks should formally recognize these ecological achievements as a “legal ecological legacy” for future descendants. Such institutional designs can transform abstract, cross-temporal environmental responsibilities into concrete, policy-endorsed legacy motivations.

Third, governmental decision-making processes should institutionalize mechanisms that represent future generations to mitigate the intergenerational dilemma. Drawing upon the principles of “future design,” governments should provide a formal voice for future generations within legal and institutional frameworks. We suggest that for all major regional planning, infrastructure construction, or resource extraction projects, a mandatory “future-generation impact assessment” phase should be integrated into the environmental impact assessment (EIA) process. Furthermore, establishing statutory “future generation representative” seats within environmental protection committees could facilitate institutionalized role-playing. This would compel current decision-makers to integrate the needs of descendants living a century from now into their cognitive frameworks, effectively addressing intergenerational short-sightedness at its source.

### 4.5. Limitations and Future Directions

Although this study provides valuable theoretical and practical insights, several limitations should be acknowledged.

Sample representativeness and omitted variable bias: The recruitment of participants in this study relied primarily on an online platform, introducing potential selection bias and resulting in a sample that skews significantly younger than the general population. Given the growing proportion of older adults in the global demographic, this demographic may possess distinct environmental attitudes, varying degrees of psychological ownership of nature, or fundamentally different legacy motivations compared to younger cohorts. Consequently, the generalizability of the psychological mechanisms explored in this study to older generations remains uncertain.

Furthermore, pro-environmental decision-making is inherently influenced by numerous sociocultural factors. The current study did not comprehensively measure or control for several key variables, including parenthood, political ideology, geographic environment, and socioeconomic status. For instance, parenthood may intrinsically heighten legacy motivation, whereas socioeconomic status could alter an individual’s perceived financial cost during intergenerational dilemmas. By isolating the specific psychological pathways of psychological ownership of nature and legacy motivation, our proposed framework inevitably presents a simplified model of these behaviors; the actual mechanisms driving pro-environmental decision-making are undoubtedly more intricate. Future research should employ stratified sampling techniques or conduct targeted surveys among older populations. Moreover, integrating the aforementioned demographic and sociopolitical variables as covariates in statistical analyses will be crucial to verify the robustness and cross-age applicability of these findings, thereby providing a more accurate and comprehensive account of the mechanisms driving pro-environmental decision-making.

Ecological validity and cost considerations of the experimental paradigm: The current study primarily employed an abstract intergenerational public goods game (IGG) within a laboratory setting; the simplified nature of this environment inherently limits its ecological validity. More critically, the experimental decisions merely involved allocating endowed tokens. This represents a low-cost scenario devoid of real effort or significant financial loss, which fails to adequately reflect the substantial costs frequently associated with real-world pro-environmental behaviors. Given that high costs typically inhibit pro-environmental choices, future research should design experiments that explicitly incorporate cost variables. For instance, subsequent studies could integrate a real effort task (RET) into the IGG paradigm, requiring participants to complete cognitive tasks of varying duration and difficulty (i.e., high versus low effort costs) to earn the resources they intend to allocate to the collective account. Alternatively, experiments could manipulate the actual monetary exchange rate of the donated tokens. Such designs would rigorously test how varying behavioral or financial costs influence the relationships among psychological ownership of nature, legacy motivation, and pro-environmental decision-making in intergenerational dilemmas.

Temporal limitations of experimental manipulations and lack of longitudinal design: The experimental design of this study relies heavily on short-term situational priming (e.g., writing tasks) to manipulate natural psychological ownership and legacy motivation; however, the efficacy and efficiency of such interventions require further enhancement. Due to the cross-sectional nature of the data, the current experimental structure precludes a definitive determination of whether these briefly induced psychological states and their subsequent impacts on pro-environmental decision-making can be sustained over the long term and eventually transform into habitual behaviors. Future research is encouraged to refine experimental intervention methods to achieve priming with higher ecological validity. Moreover, it is imperative to employ longitudinal tracking designs or long-term field experiments to evaluate the enduring effects of these variables. Additionally, longitudinal studies could incorporate “parenthood status” as a moderating variable to investigate whether the transition to parenthood alters the efficiency with which natural psychological ownership is converted into legacy motivation.

Lack of rigorous statistical verification of causal relationships: The present study has certain statistical limitations in establishing rigorous causal relationships among variables. While the experimental components utilized manipulations to assist in inferring causal direction, the cross-sectional survey data relied primarily on standard regression and mediation analyses. These methods fundamentally establish correlational pathways rather than strict causal links. Consequently, it is impossible to entirely rule out potential issues such as reverse causality or unobserved confounding variables within the mathematical framework of this study. Future research should employ advanced analytical techniques—such as instrumental variable (IV) analysis, propensity score matching (PSM), or cross-lagged panel models—to rigorously evaluate and empirically verify the causal mechanisms underlying psychological ownership of nature, legacy motivation, and pro-environmental behavior.

Generalizability boundaries of cultural context: Both the theoretical framework and empirical testing of this research are situated within the context of Chinese collectivist culture. Given the specific traits of psychological ownership, future research needs to cross cultural boundaries to examine whether natural psychological ownership might evoke territoriality or manifest more negative effects in individualistic cultures that emphasize exclusive ownership.

## 5. Conclusions

This study yielded the following conclusions through surveys and experiments: (1) Collective psychological ownership of nature promotes pro-environmental decision-making in intergenerational dilemmas. Specifically, individuals with higher psychological ownership of nature are more likely to make pro-environmental choices when facing intergenerational conflicts of interest. (2) Legacy motivation serves as a key driver for intergenerational cooperation; individuals with a higher level of impact legacy motivation demonstrate a significantly greater propensity for pro-environmental decision-making. (3) Legacy motivation mediates the effect of psychological ownership of nature on pro-environmental decision-making. Specifically, heightened psychological ownership of nature catalyzes impact legacy motivation which, in turn, facilitates sustainable decision-making for future generations.

## Figures and Tables

**Table 1:** Descriptive statistics and correlation analysis of variables (*N* = 505).

	*M*	*SD*	1	2	3	4	5
Psychological Ownership of Nature	5.86	0.95	-				
Legacy Motivation	5.05	1.08	0.37 ***	-			
Impact Legacy Motivation	5.46	0.97	0.47 ***	0.82 ***	-		
Reputation Legacy Motivation	4.64	1.47	0.23 ***	0.92 ***	0.53 ***	-	
Pro-environmental Decision-making	5.52	2.62	0.16 ***	0.24 ***	0.28 ***	0.17 ***	-

Note: *** *p* < 0.001.

**Table 2:** Regression analysis results of legacy motivation (*N* = 505).

	Pro-Environmental Decision-Making in Intergenerational Dilemma		Legacy Motivation		Pro-Environmental Decision-Making in Intergenerational Dilemma	
	*β*	*t*	*β*	*t*	*β*	*t*
Psychological Ownership of Nature	0.16	3.58 ***	0.37	9.09 ***	0.07	1.59
Legacy Motivation					0.23	4.79 ***
Age	0.03	0.68	0.02	0.60	0.02	0.57
Gender	0.02	0.52	−0.19	−4.66 ***	0.07	1.50
*R* ^2^	0.03		0.17		0.07	
*F*	4.61 **		34.77 ***		9.34 ***	

Note: ** *p* < 0.01, *** *p* < 0.001.

**Table 3:** Mediation effect test results of legacy motivation (*N* = 505).

Path	Effect	Standardized Estimate	Boot SE	Bootstrap 95% CI	
				Lower	Upper
Psychological ownership of nature → legacy motivation → pro-environmental decision-making in intergenerational dilemma	Total effect	0.16	0.04	0.07	0.24
	Direct effect	0.07	0.05	−0.02	0.17
	Indirect effect	0.08	0.02	0.05	0.13
Psychological ownership of nature → impact legacy motivation (M1)/reputation legacy motivation (M2) → pro-environmental decision-making in intergenerational dilemma	Total effect	0.16	0.04	0.07	0.24
	Direct effect	0.03	0.05	−0.06	0.13
	Total indirect effect	0.13	0.03	0.07	0.19
	Indirect effect M1	0.11	0.03	0.06	0.18
	Indirect effect M2	0.01	0.01	−0.01	0.04
	Comparison of indirect effects	0.10	0.04	0.04	0.18

**Table 4:** One-way ANOVA results of the impact of psychological ownership of nature on each variable (*N* = 180).

Variable	Psychological Ownership of Nature (*M* ± *SD)*		*F*	*Sig.*	*ηp* ^2^
	Experimental Group (*n* = 90)	Control Group (*n* = 90)			
Impact legacy motivation	5.99 ± 0.47	5.78 ± 0.71	5.49	0.020	0.030
Reputation legacy motivation	5.40 ± 0.90	5.28 ± 1.05	0.64	0.424	0.004
Pro-environmental decision-making in intergenerational dilemma	6.74 ± 1.50	6.30 ± 1.47	4.02	0.047	0.022

**Table 5:** One-way ANOVA results on the effect of legacy motivation on pro-environmental decision-making in intergenerational dilemma (*N* = 230).

Variable	Legacy Motivation (*M* ± *SD)*			*F*	*Sig.*	*ηp* ^2^
	Impact Experimental Group (*n* = 79)	Reputation Experimental Group (*n* = 75)	Control Group (*n* = 76)			
Pro-environmental decision-making in intergenerational dilemma	6.48 ± 1.58	6.01 ± 1.70	5.67 ± 1.16	5.74	0.004	0.048

## Data Availability

The data in this study belongs to the research group’s independently collected database, and it can be obtained from the authors upon reasonable request.
